# Increased Risk of Sudden Cardiac Arrest in Obstructive Pulmonary Disease: A Case-Control Study

**DOI:** 10.1371/journal.pone.0065638

**Published:** 2013-06-06

**Authors:** Miriam Jacoba Warnier, Marieke Tabo Blom, Abdennasser Bardai, Jocelyn Berdowksi, Patrick Cyriel Souverein, Arno Wilhelmus Hoes, Frans Hendrik Rutten, Anthonius de Boer, Rudolph Willem Koster, Marie Louise De Bruin, Han Liong Tan

**Affiliations:** 1 Division of Pharmacoepidemiology and Clinical Pharmacology, Utrecht Institute of Pharmaceutical Sciences, Utrecht University, Utrecht, The Netherlands; 2 Julius Center for Health Sciences and Primary Care, University Medical Center Utrecht, Utrecht, The Netherlands; 3 Heart Center, Academic Medical Center, University of Amsterdam, Amsterdam, The Netherlands; 4 Interuniversity Cardiology Institute Netherlands, Utrecht, The Netherlands; 5 Department of Cardiology, Academic Medical Center, University of Amsterdam, Amsterdam, The Netherlands; CUNY, United States of America

## Abstract

**Background:**

We aimed to determine whether (1) patients with obstructive pulmonary disease (OPD) have an increased risk of sudden cardiac arrest (SCA) due to ventricular tachycardia or fibrillation (VT/VF), and (2) the SCA risk is mediated by cardiovascular risk-profile and/or respiratory drug use.

**Methods:**

A community-based case-control study was performed, with 1310 cases of SCA of the ARREST study and 5793 age, sex and SCA-date matched non-SCA controls from the PHARMO database. Only incident SCA cases, age older than 40 years, that resulted from unequivocal cardiac causes with electrocardiographic documentation of VT/VF were included. Conditional logistic regression analysis was used to assess the association between SCA and OPD. Pre-specified subgroup analyses were performed regarding age, sex, cardiovascular risk-profile, disease severity, and current use of respiratory drugs.

**Results:**

A higher risk of SCA was observed in patients with OPD (n = 190 cases [15%], 622 controls [11%]) than in those without OPD (OR adjusted for cardiovascular risk-profile 1.4 [1.2–1.6]). In OPD patients with a high cardiovascular risk-profile (OR 3.5 [2.7–4.4]) a higher risk of SCA was observed than in those with a low cardiovascular risk-profile (OR 1.3 [0.9–1.9]) The observed SCA risk was highest among OPD patients who received short-acting β2-adrenoreceptor agonists (SABA) or anticholinergics (AC) at the time of SCA (SABA OR: 3.9 [1.7–8.8], AC OR: 2.7 [1.5–4.8] compared to those without OPD).

**Conclusions:**

OPD is associated with an increased observed risk of SCA. The most increased risk was observed in patients with a high cardiovascular risk-profile, and in those who received SABA and, possibly, those who received AC at the time of SCA.

## Introduction

Sudden cardiac arrest (SCA) most often causes sudden death and is the most common direct cause of death in Western [1] and developing [2] societies. Given the dismal survival rate of SCA, [3], [4] identification of patients at risk is crucial to develop preventive measures. Signals have emerged that patients with obstructive pulmonary disease (OPD: asthma and chronic obstructive pulmonary disease [COPD]) do not only have a worse outcome after SCA, [5] but are also at increased risk for the occurrence of SCA. [6] This may be due to an increased risk of concomitant cardiovascular disease [7], as OPD and cardiovascular disease share risk factors and disease pathways, e.g., smoking (in COPD) and inflammation. [8], [9] Accordingly, OPD is associated with a higher risk of cardiac arrhythmias and cardiovascular mortality. [6] Alternatively, increased SCA risk in OPD may stem from drugs used to treat OPD (‘respiratory drugs’). [10] In particular, inhaled short-acting or long-acting β2-adrenoreceptor agonists (SABA, LABA) and anticholinergics (AC) have attracted suspicion, but evidence is conflicting. [11], [12].

Reports on SCA often use a practical but inaccurate definition of sudden death: witnessed natural death <1 hour of onset of acute symptoms, or unwitnessed unexpected death of someone seen in a stable medical condition <24 hours previously. [13] This may cause misclassification, e.g., by inclusion of unwitnessed respiratory failure. Confirmation that SCA was present requires electrocardiogram (ECG) documentation of ventricular tachycardia or ventricular fibrillation (VT/VF), the predominant causative arrhythmias of SCA. The first aim of the present study was therefore to establish whether OPD is associated with an increased risk of SCA with ECG-documented VT/VF. Secondly, we sought to identify subgroups of OPD patients at greatest observed risk, focusing on the possible roles of cardiovascular risk-profile and use of respiratory drugs.

## Methods

### Ethics Statement

The AmsteRdam REsuscitation Study (ARREST) was conducted according to the principles expressed in the Declaration of Helsinki. Written informed consent was obtained from all participants who survived SCA. The Ethics Committee of the Academic Medical Center Amsterdam approved the use of data from patients who did not survive SCA, and approved this study.

### Setting and Study Design

We performed a community-based case-control study. Cases were SCA patients from the ARREST database. Each case was matched to five controls without SCA by age, sex and index date (date of SCA in cases) drawn from the PHARMO record linkage system (www.PHARMO.nl).

ARREST is specifically designed to study the causes and outcome of SCA in the community (out-of-hospital). All individuals who suffer SCA in the North Holland province of the Netherlands (>2.4 million inhabitants) are included. The ARREST study protocol is described in detail elsewhere. [14] In short, a data collection infrastructure is used to record all SCA parameters, from ambulance dispatch to discharge from the hospital or until death. ECG recordings from the ambulance monitor/defibrillator or automated external defibrillator are used to determine whether VT/VF occurred. Cases were patients older than 40 years with *incident* SCA; i.e. those with a first diagnosis of SCA, with ECG-documented VT/VF. Patients were excluded when cardiac arrest was caused by trauma, drowning, intoxication, or other unequivocal non-cardiac causes. Patients in whom only asystole (but no VT/VF) was recorded were excluded, because we could not ensure that cardiac arrest stemmed from cardiac causes, as asystole is the end stage of any cardiac arrest, and may be due to non-cardiac causes (e.g., respiratory failure). [15] Of each case, complete medication history of the year before SCA was retrieved by contacting the patient’s pharmacy. Data for the current study were retrieved from July 2005 to December 2008.

The PHARMO database includes drug-dispensing records from community pharmacies of >3 million community-dwelling inhabitants in the Netherlands. The catchment area covers about 12% of the total population of the Netherlands, and is representative of the total population. Since nearly all patients in the Netherlands are registered at a single community pharmacy, independent of prescriber, pharmacy records are essentially complete. [16].

Because it is virtually impossible to disentangle the effects of OPD from those of respiratory drugs, as nearly all OPD patients use such drugs, and those more severely affected generally use more drugs (often from multiple drug classes), [17], [18] OPD patients among cases and controls were identified by using the prescription of respiratory medication as proxy; patients were considered to suffer from OPD if they had at least two prescriptions of any drug with Anatomical Therapeutic Chemical classification (ATC) code R03 (drugs for obstructive airway diseases, either oral or inhaled), within one year prior to the index date.

### Covariates

As the association between OPD and SCA may be confounded by patient and other characteristics associated with both the presence of OPD and the risk of SCA, we studied the influence of various covariates on the calculated associations. Potential confounders studied were high cardiovascular risk-profile, diabetes mellitus, and current use of antiarrhythmic drugs or non-antiarrhythmic QTc prolonging drugs. High cardiovascular risk-profile was defined as the use of any of the following drugs within 6 months before index date: β-adrenoreceptor blockers, calcium channel antagonists, angiotensin-converting enzyme inhibitors, diuretics, angiotensin-II receptor blockers, nitrates, platelet aggregation inhibitors, and/or statins. Diabetes mellitus was defined by use of anti-diabetics within six months before index date. Anti-arrhythmic drugs were Vaughan-Williams class I or III antiarrhythmic drugs (Table S1) [19]. Non-antiarrhythmic QT prolonging drugs were class 1 or 2 QTc-prolonging drugs according to the Arizona Center for Education & Research on Therapeutics (Table S1). Drug use was defined as current if the index date fell between the prescription date and the end of the prescription period (extended with 10% after the prescribed duration to account for irregular drug use). We defined two age-categories: <65 years, and ≥65 years. The number of different respiratory drugs used in the six-month period before index date was used as a proxy for OPD severity. [17], [18] OPD patients were grouped according to the current use of respiratory drugs: SABA, LABA, AC, inhaled corticosteroids (ICS), alone or in combinations (mutually exclusive categories). In the Netherlands, OPD is treated almost exclusively with inhaled respiratory medications. Therefore oral medications, such as systemic β2-adrenoreceptor agonists, xanthines, or chronic systemic corticosteroid use, were not included in the analyses. In the Netherlands, medications used to treat OPD are not available over-the-counter.

### Data Analyses

Differences in baseline characteristics were examined with chi-square tests or t-tests. Conditional logistic regression analysis was used to examine the association between SCA and OPD, with adjustment for three sets of confounders: 1) all potential confounders, 2) all covariates that were univariately associated with SCA (p<0.05), 3) all covariates that were univariately associated with SCA and that changed the beta-coefficient of the association between OPD and SCA by ≥5%. As logistic regression analyses were performed, odds ratios were calculated, which can be interpreted as a risk ratio in case of adequate sampling of controls from the study base. Stratified analyses were performed regarding age category, sex, and cardiovascular risk-profile. The presence of interaction on a multiplicative scale between OPD and cardiovascular risk-profile, was estimated by including the cross-product of the two factors as a variable in the model. The presence of interaction on an additive scale between OPD and high cardiovascular risk-profile was estimated by determining the synergy index. [20] Subgroup analyses were performed according to disease severity of OPD, and (combinations of) types of current use of respiratory drugs. All data were analysed using the statistical software package SPSS (SPSS for Windows, version 18.0, SPSS Inc.).

## Results

**Figure 1:**
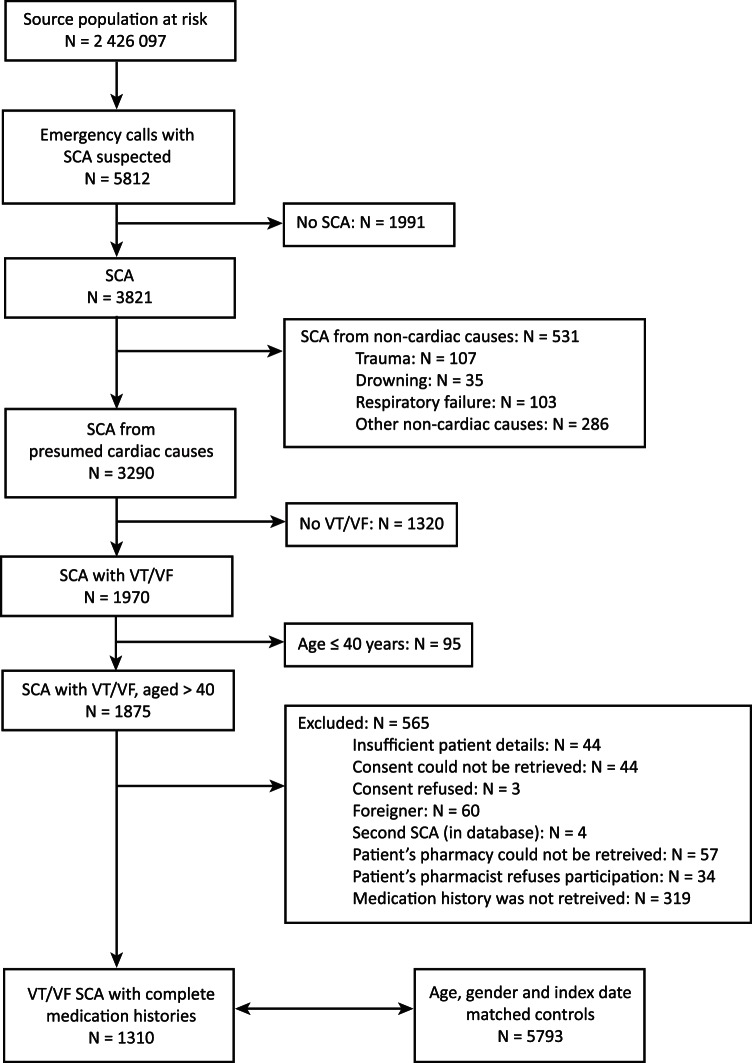
Flow chart. The source region had a population of 2 426 097 people in 2007 [Netherlands Statistics. http://statline.cbs.nl/. Accessed May 15, 2010].

During the study period, 3821 instances of cardiac arrest were recorded, of which 1875 cases had ECG-documented VT/VF and were aged >40 years. We excluded 565 patients (figure 1; excluded vs. included patients: mean age 65.0 [SD 12.2] vs. 67.1 years [SD 12.6], p<0.001; male sex 80% vs. 78%, p = 0.237). The study population consisted of 1310 SCA cases; these were matched with 5793 controls without SCA. Characteristics of cases and controls are presented in Table 1. The mean age was 67.1 (SD 12.2, range: 41–99) years, and 78% were male.

**Table 1:** Baseline characteristics of the study population.

Baseline characteristics	Cases	Controls	p-value
	N = 1310	N = 5793	
Mean age (years, standard deviation)	67.1 (12.2)	67.0 (12.2)	n/a
Age group in years			
<65	546 (42%)	2421 (42%)	
≥65	764 (58%)	3372 (58%)	n/a
Male sex	1015 (78%)	4502 (78%)	n/a
Comorbidities			
High cardiovascular risk-profile^4^	929 (71%)	3049 (53%)	<0.001
Diabetes mellitus^5^	232 (18%)	621 (11%)	<0.001
Current use of concomitant medication^1^			
Antiarrhythmic drugs^6^	60 (4%)	182 (3%)	0.009
β-adrenoreceptor blockers	463 (35%)	1134 (20%)	<0.001
Non-antiarrhythmic QT prolonging drugs class 1	21 (2%)	64 (1%)	0.134
Non-antiarrhythmic QT prolonging drugs class 2	35 (3%)	151 (3%)	0.891
Obstructive pulmonary disease	190 (15%)	622 (11%)	<0.001
Current β-adrenoreceptor blocker-use in OPD patients	70 (37%)	120 (19%)	<0.001
Current use of inhaled respiratory drugs^1^			
Inhaled short-acting β2-adrenoreceptor agonists	62 (5%)	51 (0.9%)	<0.001
Inhaled long-acting β2-adrenoreceptor agonists	78 (6%)	127 (2%)	<0.001
Inhaled anticholinergics	78 (6%)	102 (2%)	<0.001
Inhaled corticosteroids	97 (7%)	205 (4%)	<0.001
Other drugs used to treat OPD			
Systemic β2-adrenoreceptor agonists^2^	3 (0.2%)	4 (0.1%)	0.096
Xanthines^2^	10 (0.8%)	31 (0.5%)	0.325
Chronically used systemic corticosteroids^3^	18 (1.4%)	93 (1.6%)	0.542

Data are number (%) unless otherwise indicated. OPD: obstructive pulmonary disease.

1 Drug use at index date.

2 Drug use at index date, or within six months prior to index date.

3 Use of systemic corticosteroids with a duration of 90 days or more.

4 Use of any of the following drugs: β-adrenoreceptor blockers, calcium channel antagonists, angiotensin converting enzyme inhibitors, diuretics, angiotensin-II receptor blockers, nitrates, platelet aggregation inhibitors, and statins, within six months prior to index date.

5 Use of anti-diabetics within six months prior to index date.

6 Class I and III antiarrhythmic drugs and non-antiarrhythmic drugs with (possible) risk of QT prolongation (Table S1).

OPD was more prevalent in cases (15%) than controls (11%, p<0.001). OPD was independently associated with an increased observed risk of SCA (crude OR 1.4 [1.2–1.7]). The three different models used to adjust for confounding resulted in similar ORs (Table 2).

**Table 2:** Determinants of risk of sudden cardiac arrest.

Outcome	UnadjustedOR (95%CI)	AdjustedOR^1^ (95%CI)	AdjustedOR^2^ (95%CI)	AdjustedOR^3^ (95%CI)
Obstructive pulmonary disease	1.4 (1.2–1.7)	1.4 (1.1–1.6)	1.4 (1.1–1.6)	1.4 (1.2–1.6)
High cardiovascular risk-profile^4^	2.5 (2.2–2.9)	2.3 (2.0–2.7)	2.3 (2.0–2.7)	2.5 (2.2–2.9)
Diabetes mellitus^5^	1.8 (1.5–2.1)	1.5 (1.2–1.7)	1.5 (1.2–1.7)	
Use of antiarrhythmic drugs^6^	1.5 (1.1–2.0)	1.2 (0.9–1.6)	1.2 (0.9–1.6)	
Non-antiarrhythmic QT prolonging drugs class 1^6^	1.4 (0.8–2.3)	1.2 (0.7–2.0)		
Non-antiarrhythmic QT prolonging drugs class 2^6^	1.0 (0.7–1.5)	1.0 (0.7–1.4)		

CI: confidence interval, OR: odds ratio.

1 Adjusted for all potential confounders.

2 Adjusted for all covariates that were univariately associated with sudden cardiac arrest.

3 Adjusted for all covariates that were univariately associated with sudden cardiac arrest and changed the beta with at least 5%.

4 Use of any of the following drugs: β-adrenoreceptor blockers, calcium channel antagonists, angiotensin converting enzyme inhibitors, diuretics, angiotensin-II receptor blockers, nitrates, platelet aggregation inhibitors, and/or statins, within six months prior to index date.

5 Use of anti-diabetics within six months prior to index date.

6 Class I and III antiarrhythmic drugs and non-antiarrhythmic drugs with (possible) risk of QT prolongation. (Table S1).

Stratification according to sex showed a stronger effect of OPD in women (OR_adj_ 1.8 [1.3–2.6]) than in men (OR_adj_ 1.3 [1.03–1.6], Table 3). The increase in observed SCA risk associated with OPD was slightly stronger in OPD patients younger than 65 (OR_adj_ 1.6 [1.2–2.3] than in OPD patients of 65 years or older (OR_adj_ 1.3 [1.03–1.6], Table 3).

**Table 3:** Obstructive pulmonary disease and the risk of sudden cardiac arrest stratified by age group, sex and cardiovascular risk profile^1^.

Outcome		Cases	Controls		Unadjusted	Adjusted OR^2^ (95% CI)
		N = 1310	N = 5793		OR (95% CI)	
*By age group (years)*						
<65 with OPD		58/546 (11%)	163/2421 (7%)		1.6 (1.2–2.3)	1.6 (1.2–2.3)
≥65 with OPD		132/764 (17%)	459/3372 (14%)		1.3 (1.1–1.6)	1.3 (1.03–1.6)
*By sex*						
Women with OPD		51/295 (17%)	124/1291 (10%)		2.0 (1.3–2.8)	1.8 (1.3–2.6)
Men with OPD		139/1015 (14%)	498/4502 (11%)		1.3 (1.04–1.6)	1.3 (1.03–1.6)
*By cardiovascular risk-profile*						
No OPD	Low risk profile	342 (26%)		2505 (43%)	Reference	n/a
	High risk profile	778 (59%)		2666 (46%)	2.5 (2.1–2.9)	n/a
OPD	Low risk profile	39 (3%)		239 (4%)	1.3 (0.9–1.9)	n/a
	High risk profile	151 (12%)		383 (7%)	3.5 (2.7–4.4)^3^	n/a

Data are number (%). CI: confidence interval, CVD: cardiovascular disease, N: number, n/a: not applicable, OPD: obstructive pulmonary disease, OR: odds ratio.

1 Use of β- adrenoreceptor blockers, calcium channel antagonists, angiotensin converting enzyme inhibitors, diuretics, angiotensin-II receptor blockers, nitrates, platelet aggregation inhibitors, and/or statins within six months prior to index date.

2 Adjusted for cardiovascular risk profile.

3 Interaction on a multiplicative scale: OR 1.1 (0.7–1.6), on an additive scale: synergy index 1.4 (0.7–2.6).

When we studied cardiovascular risk-profile in detail, we found that SCA risk was observed to be most elevated in OPD patients with a high cardiovascular risk-profile (OR 3.5 [2.7–4.4]), and less so in patients without OPD, but with a high cardiovascular risk-profile (OR 2.5 [2.1–2.9]) or in OPD patients with a low cardiovascular risk-profile (OR 1.3 [0.9–1.9]). No significant interaction between cardiovascular risk-profile and OPD was observed on a multiplicative scale, nor on an additive scale. The observed SCA risk increased in parallel with OPD severity: compared to patients without OPD, the observed SCA risk was more elevated in patients with very severe OPD (OR_adj_ 1.8 [1.2–2.7]) than in patients with moderate OPD (OR_adj_ 1.4 [1.1–1.7], Table 4).

**Table 4:** The risk of sudden cardiac arrest in obstructive pulmonary disease categorized in subgroups by disease severity and current use of respiratory medication. Categories are mutually exclusive.

Outcome		Cases	Controls	Unadjusted OR (95% CI)	Adjusted OR^1^ (95% CI)
		N = 1310	N = 5793		
*Disease severity of OPD* 2					
No OPD		1120 (86%)	5171 (89%)	Reference	Reference
OPD	Mild (0 drugs)	12 (1%)	61 (1%)	0.9 (0.5–1.7)	0.9 (0.5–1.7)
	Moderate (1–2 drugs)	99 (8%)	334 (6%)	1.4 (1.1–1.7)	1.4 (1.1–1.7)
	Severe (3 drugs)	45 (3%)	147 (3%)	1.4 (1.01–2.0)	1.3 (0.9–1.9)
	Very severe (>3 drugs)	34 (3%)	80 (1%)	1.9 (1.3–2.9)	1.8 (1.2–2.7)
*Current use of respiratory drugs*					
No OPD		1120 (86%)	5171 (89%)	Reference	Reference
OPD	No SABA, AC, LABA or ICS	52 (4%)	358 (6%)	0.7 (0.5–0.9)	0.7 (0.5–0.9)
	SABA only	12 (0.9%)	13 (0.2%)	4.1 (1.9–9.0)	3.9 (1.7–8.8)
	LABA only	2 (0.2%)	5 (0.1%)	1.7 (0.3–9.0)	1.8 (0.3–9.2)
	AC only	19 (2%)	30 (0.5%)	2.8 (1.6–5.0)	2.7 (1.5–4.8)
	ICS only	11 (0.8%)	78 (1.3%)	0.6 (0.3–1.2)	0.7 (0.4–1.3)
	SABA+AC	8 (0.6%)	10 (0.2%)	3.5 (1.4–8.9)	2.6 (1.02–6.7)
	ICS+LABA	26 (2%)	57 (1.0%)	2.0 (1.3–3.3)	2.0 (1.2–3.2)
	SABA+LABA and/or ICS	14 (1%)	11 (0.2%)	5.5 (2.5–12.1)	5.3 (2.4–12.0)
	AC+LABA and/or ICS	23 (2%)	46 (0.8%)	2.3 (1.4–3.8)	2.0 (1.2–3.4)
	SABA+AC+LABA and/or ICS	23 (2%)	14 (0.2%)	7.9 (3.9–16.0)	7.6 (3.7–15.6)

Data are number (%) or odds ratios (95% confidence interval).

ATC: Anatomical Therapeutic Chemical classification system, AC: anticholinergics, CI: confidence interval, ICS: inhaled corticosteroids, LABA: long-acting β2-adrenoreceptor agonists, N: number, OPD: obstructive pulmonary disease, OR: odds ratio, SABA: short-acting β2-adrenoreceptor agonists.

1 Adjusted for concomitant cardiovascular disease.

2 Number of different respiratory drugs used (ATC code R03) in the six-month period before index date. Patients with mild OPD are patients who received at least two prescriptions of any drug ATC code R03 (drugs for obstructive airway diseases), within one year prior to the index date, but who did not use any of these drugs in the six-month period before index date.

Analysis of current use of respiratory drug revealed that increased SCA risk was associated with the use of SABA only (OR_adj_ 3.9 [1.7–8.8]) or AC only (OR_adj_ 2.7 [1.5–4.8]), but not ICS only, while use of LABA only was too rare to draw any conclusions (Table 4). Use of SABA or AC in combination with other respiratory drugs was also associated with increased SCA risk. Patients who used both SABA and AC, in combination with LABA and/or ICS, had the highest observed SCA risk (OR_adj_ 7.6 [3.7–15.6], Table 4). SCA risk associated with SABA or AC use (alone or in combination with other respiratory drugs) was particularly elevated in the presence of a high cardiovascular risk-profile (SABA: 45 cases [3%], 33 controls [0.6%], OR_adj_ 6.0 [3.8–9.5], AC: 61 cases [5%], 80 controls [1%], OR_adj_ 3.5 [2.4–4.9]).

## Discussion

This is the first study to show that OPD is associated with a 40% increased risk of ECG-confirmed SCA. Multiple analytical approaches were applied to account as much as possible for confounding. These analyses consistently demonstrated a statistically significantly increased observed risk of SCA in OPD patients, with an OR of 1.4. This provides some evidence of the robustness of our findings, but residual confounding cannot be completely ruled out, because of potential misclassification in some of our measurements. The increase in observed SCA risk is most pronounced in OPD patients with a high cardiovascular risk-profile. Use of SABA increases SCA risk among OPD patients, particularly in patients with a high cardiovascular risk-profile: in these patients a six-fold increased SCA risk was observed compared to patients without OPD.

The observed increased risk of SCA in OPD patients may be, in part, due to the higher prevalence of concomitant cardiovascular disease and cardiac arrhythmias, as these are risk factors for VT/VF. However, adjusting our analyses for cardiovascular risk-profile, current use of anti-arrhythmic drugs (as a proxy for pre-existing cardiac arrhythmias), and QT prolonging drugs (which may evoke cardiac arrhythmias) did not alter the OR for SCA risk.

Still, the strongest association with SCA was observed in OPD patients with a high cardiovascular risk-profile, particularly, in those who used SABA. Other shared risk factors for OPD and cardiovascular disease may also play a role, but we were unable to assess them. Smoking, a common cause of COPD, is clearly also associated with ischemic heart disease. [21] Similarly, systemic inflammation, an important pathophysiologic mechanism in OPD, [8], [9] has emerged from epidemiologic studies as a causative factor for atherosclerosis and ischemic heart disease. [22].

To further support the role of OPD in SCA risk, we found that more severe OPD was more strongly associated with SCA risk than mild OPD. Possibly, chronic hypoxemia, more likely to occur in those with more severe OPD, may contribute to the development of cardiac arrhythmias by increasing resting heart rates, as increased heart rate is associated with increased mortality in both the general population as well as in patients with COPD. [23]–[25] These cardiac arrhythmias may cause episodes of cardiac ischemia, which is related to an increased SCA risk. [26] Paradoxically, we found that use of β-adrenoceptor blockers among OPD patients was more common in cases than controls. One would probably expect the reverse, as β-adrenoceptor blockers are the only drugs proven to prevent sudden death (possibly, in part, through heart rate slowing). [27]–[30] Moreover, these drugs have long been considered contra-indicated in COPD patients, particularly in those with severe COPD and larger SCA risk. Yet recent evidence indicates that cardio-selective β-adrenoceptor blockers are well tolerated by COPD patients, in daily practice the potential disadvantages of making COPD worse by prescribing β-adrenoceptor blockers and making and cardiac disease worse by refraining from prescribing these drug should be weighed to optimally decide on the use of β-adrenoceptor blockers in patients with OPD. [31].

Interestingly, although not statistically significantly, we found a stronger association between OPD and SCA in women than in men. This contrasts with the general population, where the observed SCA risk among women is only a third of that in men. [32] It may be speculated that this difference is, in part, mediated by concomitant, yet unrecognized and thus untreated, cardiovascular disease in women. The clinical presentation of ischemic heart disease in women differs from that in men. Symptoms of myocardial infarction are often labelled as "atypical" in women, as women are less likely to report the key symptom, chest pain or discomfort, than men. [33].

We observed that, in the studied population, patients who received SABAs at the time of SCA, especially when combined with other respiratory drugs, had a higher SCA risk than patients who received other respiratory drugs. For LABAs, this association was less clear. The association between SABA use and SCA may be explained as follows. β2-adrenoreceptor agonists act on the β2-adrenergic receptors of bronchial smooth muscle, leading to dilatation of the bronchi, which results in relief of symptomatic wheeze and dyspnoea, and improvement of lung function. [34], [35] However, β2-adrenergic receptors are also present in the heart. Here, their stimulation results in increased myocardial contractility and heart rate. [34] An elevated heart rate is associated with an increased risk of cardiac mortality in the general population [36], and in COPD patients. Moreover, β2-adrenoreceptor agonists lower serum potassium levels due to intracellular uptake of potassium by stimulation of membrane-bound Na/K-ATPase; this may cause cardiac arrhythmias. [34], [37] Finally, inhaled β2-adrenoreceptor agonists prolong the ECG QT interval, a risk factor for mortality, [38] and this effect is dose-dependent. [35].

Consequently, soon after their availability in the 1960s, concerns have emerged about potentially serious adverse effects of β2-adrenoreceptor agonists on the heart, although the currently available studies and meta-analyses yield conflicting results. The meta-analysis of Salpeter *et al.* showed that use of β2-adrenoreceptor agonists in OPD patients increases the risk of cardiovascular events. [11] In contrast, a review of Wood-Baker *et al.* concluded that there is no evidence of increased mortality associated with the use of SABAs in COPD patients, [39] and Suissa *et al.* suggest that the use of β2-adrenoreceptor agonists only increases the risk of cardiac mortality in certain administration forms. [40] An alternative explanation for the association of β2-adrenoreceptor agonist use with cardiovascular mortality is that the intensity of use, and combined use with other respiratory drugs, reflects the severity of OPD. Our study adds to this discussion by the observation that, while elevated SCA risk is, in general, associated with SABA use, this is particularly true for patients with high cardiovascular risk-profile.

In accordance with previous studies which showed that AC use was associated with an increased risk of cardiovascular death, [12] we found that OPD patients who received AC showed an overall increased risk of SCA, although to a lesser extent than patients receiving SABA. In contrast, the UPLIFT-trial did not show an increased risk of cardiovascular mortality in COPD patients using AC (tiotropium). [41] Based upon this trial, the Food and Drug Administration in 2010 concluded that current studies did not support the notion that there is an increased risk of death associated with tiotropium. [42] In accordance with this conclusion, we cannot rule out that our observed increase in SCA risk of OPD patients who receive AC may reflect COPD severity, rather than a pro-arrhythmic effect of AC *per se*, as AC are mainly used by COPD patients (not by patients with asthma), especially those with advanced disease. In any case, SCA risk associated with AC use was largest in OPD patients with a high cardiovascular risk-profile.

### Strengths and Limitations

A major strength of our study is that ARREST was specifically designed to study the determinants of SCA. This ensured that SCA diagnosis was accurate. SCA was validated by the presence of VT/VF on the ECG. This is especially important in OPD patients, because sudden death caused by cardiac arrest may easily be confused with sudden death caused by respiratory failure. [15] Another strength is that our findingsare representative for the community at large, because we studied the general population, including both urban and rural areas, and captured ∼90% of all SCA cases. [14] We only excluded eligible cases and controls because of incompleteness of data. If such incompleteness is associated with the determinant of interest (i.e. OPD) this may lead to (selection) bias. Since the main reasons of incompleteness (e.g. consent could not be retrieved or was refused, patient’s pharmacy could not be retrieved or refused participation) were highly unlikely to be related to the presence of OPD, such bias does not play an important role in our study.

A limitation is that, as this is an observational study, we were unable to completely distinguish between the effects of OPD *per se* and those of the respiratory drugs used to treat OPD. We addressed this problem by performing subgroup analyses according to disease severity, and current use of respiratory medication. Misclassification in the diagnosis of OPD could have occurred, as we defined the presence of the disease by the use of two prescriptions of respiratory drugs within one year before index date. Still, the risk of misclassification is probably limited, as these drugs are indicated exclusively for OPD, and patients with OPD who received less than two prescriptions of any respiratory drug in the past year most likely are patients with very mild disease. Besides, such a misclassification is most likely to be non-differential, i.e. not related to the risk of SCA and thus most likely results in an underestimation of the true association between OPD and SCA. Similarly, as we defined the presence of the disease by the use of medication, it was impossible to distinguish between asthma and COPD. However, we defined subgroups based on age, with the 65-years-or-younger group most likely to be asthma patients, and the 65-years-or-older group most likely to be COPD patients. Also, there may be some misclassification on drug exposure, as it is impossible to ascertain whether the patients who received a prescription for inhalation medication actually used these drugs on the index date. This may especially be true for SABA, as these are generally intermittently used as symptomatic treatment. Finally, SCA is the first clinically identified expression of heart disease in up to one-half of the instances of SCA. [43] Therefore, many important clinical measurements, such as information on smoking and alcohol use, or history of syncope or arrhythmias have never been made before the SCA episode. Smoking is an important risk factor for both COPD and cardiovascular disease, and we assume that smoking rates were higher among OPD patients than controls.

### Conclusions

We observed that the overall risk of SCA is 40% higher in patients with OPD than in patients without OPD, and we were able to identify subgroups of OPD patients in whom this risk was most elevated: those with a high cardiovascular risk-profile, those who receive SABA, and possibly those who receive AC at the time of SCA. Our findings may provide the basis for refinements in treatment strategies for OPD patients. We therefore would recommend a prospective trial to evaluate the effectiveness of more integrated pulmonary and cardiovascular care (e.g., investigations to detect previously unrecognized cardiovascular disease) in these high-risk patients.

## Supporting Information

Table S1 **Class I and III antiarrhythmic drugs, according to the classification of Vaughan-Williams^1^ (ATC code: C01B, C07AA07), and non-antiarrhythmic drugs with (possible) risk of QT prolongation according to the Arizona Center for Education & Research on Therapeutics [** http://www.azcert.org/medical-pros/drug-lists/bycategory.cfm **, accessed on December 2, 2011].**
(DOCX)
