# Supplementary material for: Increased Risk of Sudden Cardiac Arrest in Obstructive Pulmonary Disease: A Case-Control Study
Source: PLoS One. 2013 Jun 6;8(6):e65638. doi: 10.1371/journal.pone.0065638 (PMC3675036; doi:10.1371/journal.pone.0065638)
Supplement: Table S1 — Class I and III antiarrhythmic drugs, according to the classification of Vaughan-Williams1 (ATC code: C01B, C07AA07), and non-antiarrhythmic drugs with (possible) risk of QT prolongation according to the Arizona Center for Education & Research on Therapeutics [ http://www.azcert.org/medical-pros/drug-lists/bycategory.cfm , accessed on December 2, 2011]. (DOCX) [file pone.0065638.s001.docx]

**Table S1. Supplementary table.** Class I and III antiarrhythmic drugs, according to the classification of Vaughan-Williams^1^ (ATC code: C01B, C07AA07), and non-antiarrhythmic drugs with (possible) risk of QT prolongation according to the Arizona Center for Education & Research on Therapeutics [<http://www.azcert.org/medical-pros/drug-lists/bycategory.cfm>, accessed on December 2, 2011].

| **Class I and III antiarrhythmic drugs** | **Non-antiarrhythmic QT prolonging drugs class 1^1^** | **Non-antiarrhythmic QT prolonging drugs class 2^2^** |
| --- | --- | --- |
| **Class Ia** | Arsenic trioxide | Alfuzosin |
| Quinidine | Astemizole | Amantadine |
| [Procainamide](http://www.whocc.no/atc_ddd_index/?code=C01BA02&showdescription=yes) | Bepridil | Atazanavir |
| [Disopyramide](http://www.whocc.no/atc_ddd_index/?code=C01BA03&showdescription=yes) | Chloroquine | Azithromycin |
| [Sparteine](http://www.whocc.no/atc_ddd_index/?code=C01BA04&showdescription=yes) | Chlorpromazine | Chloral hydrate |
| [Ajmaline](http://www.whocc.no/atc_ddd_index/?code=C01BA05&showdescription=yes) | Cisapride | Clozapine |
| [Prajmaline](http://www.whocc.no/atc_ddd_index/?code=C01BA08&showdescription=yes) | Citalopram | Dolasetron |
| [Lorajmine](http://www.whocc.no/atc_ddd_index/?code=C01BA12&showdescription=yes) | Clarithromycin | Escitalopram |
| **Class Ib** | Domperidone | Famotidine |
| [Lidocaine](http://www.whocc.no/atc_ddd_index/?code=C01BB01&showdescription=yes) | Droperidol | Felbamate |
| [Mexiletine](http://www.whocc.no/atc_ddd_index/?code=C01BB02&showdescription=yes) | Erythromycin | Foscarnet |
| [Tocainide](http://www.whocc.no/atc_ddd_index/?code=C01BB03&showdescription=yes) | Halofantrine | Fosphenytoin |
| [Aprindine](http://www.whocc.no/atc_ddd_index/?code=C01BB04&showdescription=yes) | Haloperidol | Gatifloxacin |
| **Class Ic** | Levomethadyl | Gemifloxacin |
| [Propafenone](http://www.whocc.no/atc_ddd_index/?code=C01BC03&showdescription=yes) | Mesoridazine | Granisetron |
| [Flecainide](http://www.whocc.no/atc_ddd_index/?code=C01BC04&showdescription=yes) | Methadone | Indapamide |
| [Lorcainide](http://www.whocc.no/atc_ddd_index/?code=C01BC07&showdescription=yes) | Moxifloxacin | Isradipine |
| [Encainide](http://www.whocc.no/atc_ddd_index/?code=C01BC08&showdescription=yes) | Pentamidine | Lapatinib |
| **Class III** | Pimozide | Levofloxacin |
| [Amiodarone](http://www.whocc.no/atc_ddd_index/?code=C01BD01&showdescription=yes) | Probucol | Lithium |
| [Bretylium tosilate](http://www.whocc.no/atc_ddd_index/?code=C01BD02&showdescription=yes) | Sparfloxacin | Moexipril/HCTZ |
| [Bunaftine](http://www.whocc.no/atc_ddd_index/?code=C01BD03&showdescription=yes) | Terfenadine | Nicardipine |
| [Dofetilide](http://www.whocc.no/atc_ddd_index/?code=C01BD04&showdescription=yes) | Thioridazine | Nilotinib |
| [Ibutilide](http://www.whocc.no/atc_ddd_index/?code=C01BD05&showdescription=yes) | Vandetanib | Octreotide |
| [Tedisamil](http://www.whocc.no/atc_ddd_index/?code=C01BD06&showdescription=yes) |  | Ofloxacin |
| [Dronedarone](http://www.whocc.no/atc_ddd_index/?code=C01BD07&showdescription=yes) |  | Ondansetron |
| [Sotalol](http://www.whocc.no/atc_ddd_index/?code=C07AA07) |  | Oxytocin |
| [Moricizine](http://www.whocc.no/atc_ddd_index/?code=C01BG01&showdescription=yes) |  | Paliperidone |
| [Cibenzoline](http://www.whocc.no/atc_ddd_index/?code=C01BG07&showdescription=yes) |  | Perflutren lipid microspheres |
| [Vernakalant](http://www.whocc.no/atc_ddd_index/?code=C01BG11&showdescription=yes) |  | Quetiapine |
|  |  | Ranolazine |
|  |  | Risperidone |
|  |  | Roxithromycin |
|  |  | Sertindole |
|  |  | Sunitinib |
|  |  | Tacrolimus |
|  |  | Tamoxifem |
|  |  | Telithromycin |
|  |  | Tizanidine |
|  |  | Vardenafil |
|  |  | Venlafaxine |
|  |  | Voriconazole |
|  |  | Ziprasidone |

^1^ Drugs with Risk of Torsade de Pointes and QT prolongation

^2^ Drugs with Possible Risk of Torsades de Pointes and QT prolongation

**Reference**

1 Vaughan Williams EM. (1975) Classification of antidysrhythmic drugs. Pharmacol Ther B*.* 1: 115-138.
